# From past to present and toward the future: a mechanism-informed clinical framework for COVID-19-associated headache and post-viral pain

**DOI:** 10.3389/fneur.2026.1937278

**Published:** 2026-10-13

**Authors:** Cheng He, Yan Xue, Yao Liu, Zhou-Can Xu, Rui-Qi Zhao, Jia-Yi Sun, Jian-Feng Meng, Meng-Ruo Song, Zhi-Jun Zhang, Qun-Feng Gui

**Affiliations:** 1 Department of Neurosurgery, The Sixth Affiliated Hospital of Nantong University, Yancheng Third People's Hospital, Yancheng, Jiangsu, China; 2 Department of Human Anatomy, Medical School of Nantong University, Nantong, Jiangsu, China; 3 Nantong University, Nantong, Jiangsu, China; 4 Department of Implantology, Stomatological Hospital and Dental School of Tongji University, Shanghai Engineering Research Center of Tooth Restoration and Regeneration, Shanghai, China; 5 Jiangsu Province Key Laboratory in University for Inflammation and Molecular Drug Target, Nantong University, Nantong, Jiangsu, China

**Keywords:** COVID-19, cytokine storm, headache, long COVID, management, neuroinflammation, pathophysiology, trigeminovascular system

## Abstract

Headache is a prominent acute and persistent neurological manifestation of COVID-19, representing both as a significant clinical challenge and a valuable model for understanding the neurotropic effects of SARS-CoV-2. This review synthesizes evidence showing that the epidemiology of COVID-19 headache is distinctly shaped by younger age, female sex, and a prior history of headache disorders; its clinical phenotypes are diverse, strongly associating with chemosensory dysfunctions such as anosmia; and its core pathophysiology involves neuroinflammation (e.g., cytokine-mediated pathways), trigeminovascular activation, and sex-specific neuroendocrine-immune interactions. Although acute headache often responds to conventional analgesics, persistent cases—a major component of Post-Acute Sequelae of COVID-19 (PASC)—necessitate a multidisciplinary management approach. This contrasts with the typically self-limiting headache following vaccination. Future research should prioritize longitudinal studies to define chronicity, mechanistic investigations into sex-specific pathways, and the development of phenotype-specific, personalized therapeutic strategies.

## 1 Introduction

Headache has emerged as a prominent neurological manifestation of the novel severe acute respiratory syndrome coronavirus 2 (SARS-CoV-2) infection. During the acute phase, its reported prevalence varies widely, with prospective studies indicating a range from 28.9% to 74.6%, highlighting considerable heterogeneity across populations and study designs (1–4). Clinically, acute COVID-19 headache is frequently characterized as moderate to severe, bilateral or holocranial, and throbbing in quality, often accompanied by photophobia, phonophobia, and anosmia/ageusia, with a typical duration of 2 to 14 days following symptom onset (5). These features suggest that COVID-19-associated headache is not merely a nonspecific accompaniment to systemic viral illness, but a clinically meaningful neurological syndrome with recognizable phenotypic patterns.

A significant concern is the evolution of acute headache into a persistent condition within the post-acute sequelae of COVID-19 (PASC), commonly known as Long COVID. Early systematic reviews indicated that a substantial proportion of patients experienced headaches persisting for 60 days (16.5%), 90 days (10.6%), and even beyond 180 days (8.4%) after disease onset (3, 4, 6, 7). More recent, large-scale epidemiological evidence robustly supports this association. A major 2025 nationwide case-control study demonstrated that a pre-existing headache diagnosis was a significant risk factor for developing Long COVID, with adjusted odds ratios (OR) of 2.45 (95% CI 2.29–2.62) for women and 2.89 (95% CI 2.61–3.20) for men (8). This finding underscores headache not merely as an acute symptom but as a potential marker for heightened susceptibility to prolonged, multi-system illness. Accordingly, COVID-19 headache should be considered along a temporal continuum spanning acute infection, symptom persistence, and, in a subset of patients, progression toward chronic post-viral pain states.

Although early cohort data suggested a general decline in headache prevalence following the acute phase, contemporary research underscores that for a significant minority of patients, headache evolves into a persistent and disabling chronic condition (9). Notably, a 2025 meta-analysis reported that approximately 20% of patients with acute COVID-19 headache may develop a chronic daily headache phenotype (10). The enduring nature of this symptom constitutes a major contributor to the long-term morbidity and reduced quality of life associated with PASC, highlighting the urgent need for dedicated management strategies and further pathophysiological investigation. Importantly, the key unresolved question is not simply how often headache occurs during COVID-19, but why it resolves rapidly in some individuals while becoming persistent and treatment-refractory in others. Addressing this question requires an integrated view of host susceptibility, acute inflammatory and neurovascular triggers, and mechanisms of pain chronification.

Headache, however, represents only one facet of the broad spectrum of neurological sequelae triggered by SARS-CoV-2. The infection is associated with a wide array of other neurological manifestations, ranging from common sensory disturbances such as anosmia and ageusia to cognitive impairment (“brain fog”), dizziness, and, in severe cases, cerebrovascular events or encephalitis (11). This diverse neurological involvement marked a pivotal shift in the clinical understanding of COVID-19, reframing it from a predominantly respiratory illness to a systemic disease. Although respiratory symptoms such as fever, cough, and dyspnea remain hallmarks, the pervasive multisystemic nature of COVID-19 is now unequivocally recognized (12) (Table 1). Within this broader neurological context, headache is of particular interest because it lies at the intersection of systemic inflammation, chemosensory dysfunction, trigeminovascular activation, and post-viral symptom persistence. As such, it offers a clinically accessible and biologically informative model for understanding how SARS-CoV-2 may trigger both acute neurological symptoms and longer-term maladaptive pain states.

**Table 1:** Clinical manifestations of COVID-19-associated complications.

Complication type	Specific complication/symptom	Symptom description	Reference
Cardiovascular system	Myocarditis	Chest pain, arrhythmia, fatigue; elevated cardiac injury markers (e.g., troponin).	(69)
	Arrhythmia	Palpitations, tachycardia/bradycardia, potential syncope.	(70)
	Thrombosis-related (DVT, pulmonary embolism)	Leg swelling/pain (DVT); sudden chest pain, dyspnea, hemoptysis (pulmonary embolism).	
Immune system	Cytokine storm	Persistent high fever, tachypnea, hypotension, multi-organ failure	(12, 47, 71, 72)
	Autoimmune reactions	Arthritis (joint swelling/pain), lupus-like symptoms (rash, fever).	(69, 73)
Neurological system	Acute symptoms	Headache, anosmia/ageusia; confusion, seizures, stroke (hemiplegia, aphasia).	(74, 75)
	Peripheral neuropathy	Limb numbness, muscle weakness (e.g., Guillain-Barré Syndrome).	(76, 77)
	Long-term sequelae	Cognitive impairment (memory loss, poor concentration); anxiety, depression, PTSD.	(72, 75)
	Severe conditions	Encephalitis (high fever, convulsions, altered consciousness).	(77)
Respiratory system	Pneumonia	Cough, fever, dyspnea; CT findings: ground-glass opacities or consolidation.	(71, 78, 79)
	Acute respiratory distress syndrome (ARDS)	Severe hypoxemia, tachypnea, requiring mechanical ventilation.	(71, 78)
Digestive system	Gastrointestinal symptoms	Nausea, vomiting, diarrhea (10%-20% of patients).	(69)
Skin system	Rash	Erythema, papules, urticaria-like lesions.	(80)
	Vascular lesions	Chilblain-like lesions (COVID toes); purpura, livedo reticularis (microthrombi).	(81)
	Other skin manifestations	Dryness, pruritus, desquamation.	
Endocrine system	Aggravated diabetes	Poor glycemic control, increased risk of ketoacidosis.	(80, 82)
	Thyroid dysfunction	Fluctuating thyroid hormone levels.	
Systemic complications	Systemic inflammatory response syndrome (SIRS)	Hyper/hypothermia, tachycardia, tachypnea, leukocyte abnormalities.	(73, 83)
	Multi-organ dysfunction syndrome (MODS)	Failure of heart, lungs, liver, kidneys, etc.	(84)

In this review, we propose that COVID-19-associated headache should not be viewed as a single, uniform symptom, but rather as a spectrum disorder shaped by the interaction between epidemiological predisposition, phenotype-specific clinical expression, and multiple overlapping pathophysiological pathways. We therefore synthesize current evidence within a mechanistic and clinical framework linking host factors, acute disease biology, phenotypic heterogeneity, persistence in PASC, and implications for individualized management.

The pandemic's profound societal impact, including lockdowns and social distancing, has also contributed to a surge in mental health issues such as anxiety, depression, and sleep disturbances. These stressors, combined with the direct neurological effects of the virus, have likely exacerbated the population burden of headache disorders (Table 2). It should be noted that the 1.96% figure shown in Table 2 refers to the prevalence reported in the specific hospitalized cohort described by Dias et al. (13), based on that study's neurological case definition, rather than the overall prevalence of acute COVID-19-associated headache. Understanding the interaction between COVID-19 and headache is essential for mitigating acute and long-term consequences. The underlying mechanisms are complex and multifactorial. A prominent hypothesis involves the release of pro-inflammatory cytokines, often termed a “cytokine storm,” which sensitizes peripheral and central pain pathways (14). This inflammatory response, coupled with potential direct neuroinvasion via olfactory and trigeminal pathways, contributes to headache pathogenesis. Additional mechanisms include hypoxia from respiratory dysfunction, endothelial injury leading to vascular inflammation, and coagulopathy associated with COVID-19-related hypercoagulable states (12, 15). Notably, these headaches often show a poor response to typical analgesics and are frequently associated with anosmia and ageusia. A distinct focus of this review is the exploration of mechanisms underlying the higher headache susceptibility observed in female patients, including the roles of estrogen and the prolactin-estrogen interaction network. To improve the translational value of this review, we summarize the proposed mechanism-informed clinical framework in Box 1, which links host susceptibility, clinical phenotypes, putative mechanisms, and management implications. This schematic framework integrates predisposing and contextual factors, major clinical phenotypes, putative biological mechanisms, and practical management implications for COVID-19-associated headache. Younger age, female sex, pre-existing primary headache disorders, disease severity, post-acute/Long COVID context, and variant/vaccination-related factors may shape headache susceptibility and presentation. Clinically, headache may manifest as an acute diffuse viral-like headache, a migraine-like or tension-type-like phenotype, a red-flag presentation suggestive of secondary pathology, or a persistent post-COVID/NDPH-like phenotype. These presentations may reflect overlapping contributions from systemic inflammation, neuroimmune activation, trigeminovascular sensitization, endothelial dysfunction, autonomic dysregulation, and impaired endogenous resolution. The framework is intended to support pragmatic clinical stratification, differential diagnosis, and individualized management rather than rigid classification.

**Table 2:** Percentage of symptoms caused by COVID-19.

Symptom category	Specific symptoms	Prevalence	Reference
Respiratory symptoms	Fever	84.2%	(16)
	Cough	62%	(17)
	Fatigue or myalgia	39.4%	
	ICU admission (hospitalized)	28.8%	(18)
Gastrointestinal symptoms	Diarrhea, nausea, vomiting, abdominal pain	3.0%−40.7%	(85, 86)
	Intestinal ischemia	0.7%	(87, 88)
Neurological symptoms	Selected neurological manifestations reported in a hospitalized cohort	1.96%	(13, 89, 90)
Olfactory/Gustatory dysfunction	Loss of smell or taste	60%	(91, 92)
Cutaneous manifestations	Rash, urticaria	Reported in literature (no specific prevalence)	(93–95)
Ocular symptoms	Conjunctivitis, etc.	32%	(96, 97)

Box 1 **Mechanism-informed clinical framework for COVID-19-associated headache.**

**Figure:**
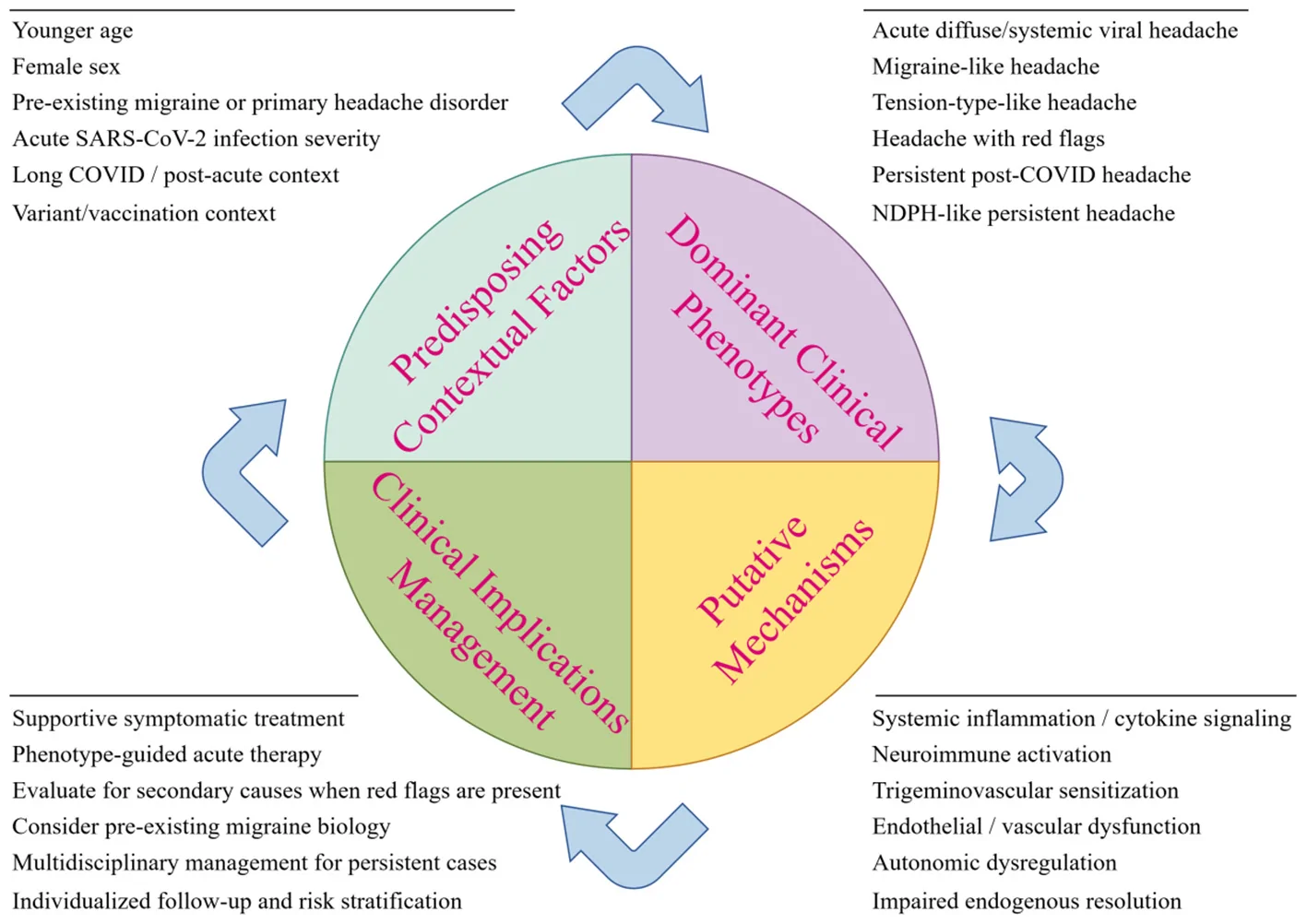

## 2 Epidemiology

Headache is a highly prevalent and diagnostically significant neurological manifestation of SARS-CoV-2 infection. Its epidemiology is heterogeneous rather than uniform, shaped by disease severity, host characteristics, pre-existing headache biology, and the risk of persistence beyond the acute phase. These epidemiological variations are clinically informative, aiding in the identification of susceptible subgroups, anticipation of phenotype variations, and formulation of hypotheses regarding chronification in PASC. Understanding these patterns is crucial for risk stratification, clinical prognosis, and the development of targeted management strategies. Representative epidemiological and clinical studies on COVID-19-associated headache are summarized in Table 3.

Table 3Key epidemiological studies of COVID-19-associated headache.

**Table:** 

Study	Design/population	Acute headache prevalence	Follow-up duration
Rocha-Filho (15)	Narrative review of published cohorts	28.9%−74.6%	Variable across included studies
Hasani et al (16)	Systematic review and meta-analysis of clinical characteristics in COVID-19	Variable across studies	Acute phase only
Canatan et al. (17)	Clinical review of pulmonary and extrapulmonary manifestations in adult patients	Headache recognized as a common extrapulmonary symptom	Acute phase only
Cao et al. (18)	Systematic review and meta-analysis of imaging and clinical features in hospitalized COVID-19 patients	Variable across included studies	Hospitalization period
Bolay et al. (47)	Mechanism-oriented review integrating early clinical observations	Broadly consistent with early cohort estimates	Acute phase only
Caronna et al. (19)	Clinical descriptive study/perspective on persistent headache after mild COVID-19	NR	Weeks to months
Mutiawati et al. (98)	Comparative clinical study of headache characteristics and headache-related quality of life in COVID-19 and non-COVID-19 patients	NR	Short-term clinical assessment
Tana et al. (20)	Review focused on Long COVID-19-associated headache syndromes	Summarized from prior studies	Variable across included studies
Hrytsenko et al. (22)	Observational study on the impact of the COVID-19 pandemic on patients with migraine	Not designed to estimate acute COVID-19 headache prevalence	Variable
Shanthanna et al. (23)	Narrative review on chronic pain consequences of the COVID-19 pandemic	Not designed to quantify acute headache prevalence	Medium- to long-term post-acute context
Finsterer et al. (24)	Review on the neuro-pathophysiological basis of COVID-19 headache	Headache summarized as a common neurological symptom	Variable
Dono et al. (21)	Case report of new daily persistent headache after SARS-CoV-2 infection	NA	Case-based follow-up
Arca and Starling (31)	Case report of treatment-refractory headache in COVID-19 pneumonia	NA	Short clinical course

**Table:** 

Study	Persistent headache prevalence	Age/sex findings	Severity association	Variant/vaccination context
Rocha-Filho (15)	Heterogeneous across studies	More frequently reported in younger patients and women in several cohorts	Often observed in mild-to-moderate COVID-19, although not limited to these groups	Mostly pre-Omicron; vaccination not systematically assessed
Hasani et al. (16)	NR	Limited stratified demographic data	Reported across severity groups	Early pandemic studies; no variant- or vaccination-specific stratification
Canatan et al. (17)	NR	Limited age- and sex-specific analysis	Reported in both ambulatory and hospitalized patients	Early pandemic; no variant/vaccination stratification
Cao et al. (18)	NR	NR	Prevalence may be influenced by hospitalized population and symptom ascertainment	Early pandemic; no vaccination data
Bolay et al. (47)	NR	Suggested possible links to female sex and migraine-like phenotype	Headache may occur even in patients without severe respiratory disease	Early first-wave era; no vaccination context
Caronna et al. (19)	Persistent headache described in distinct migraine-like scenarios	Persistence may occur in patients with prior migraine susceptibility or *de novo* phenotype	Focused on mild COVID-19 cases	Pre-vaccination or early vaccination period; variant-specific data unavailable
Mutiawati et al. (98)	NR	Evaluated headache burden and quality of life; limited generalizability for sex-stratified inference	Not primarily designed to assess severity association	Variant/vaccination context not clearly defined
Tana et al. (20)	Frequent but heterogeneous across studies	Female predominance discussed in several post-COVID cohorts	Persistent headache not restricted to severe acute infection	Multi-era literature; variant and vaccination effects remain uncertain
Hrytsenko et al. (22)	NR	Relevant to patients with pre-existing migraine susceptibility	Supports distinction between exacerbation of primary headache and new secondary headache	Pandemic-era study; variant/vaccination effects not primary focus
Shanthanna et al. (23)	Chronic pain persistence, including headache, discussed within broader post-COVID pain syndromes	Suggests biopsychosocial contributors to chronic symptom persistence	Persistence not solely explained by acute disease severity	Broad pandemic context; no firm variant/vaccination conclusions
Finsterer et al. (24)	Persistent manifestations discussed conceptually	Notes possible interaction with migraine biology and sex-related susceptibility	Mechanisms discussed across mild and severe disease spectra	Mixed pandemic phases; no firm variant/vaccination conclusions
Dono et al. (21)	NDPH-like persistence described	Case report only; not suitable for demographic inference	Can occur after non-severe infection	Early pandemic case-based evidence
Arca and Starling (31)	Persistent/refractory headache described	Case report only	Illustrates need to distinguish migraine-like headache from CNS complication	Early pandemic case-based evidence

NR, not reported; NA, not applicable.

### 2.1 Prevalence during acute infection

Headache is a common neurological and extrapulmonary manifestation of acute SARS-CoV-2 infection, although reported prevalence varies considerably across studies. This heterogeneity likely reflects differences in study setting, case ascertainment, disease severity, population characteristics, and the definition and timing of headache assessment (15–18). Headache may occur as a new symptom during infection or as an exacerbation or modification of a pre-existing primary headache disorder.

Several clinical and host-related factors may influence the occurrence and phenotype of headache during acute SARS-CoV-2 infection, including demographic characteristics, pre-existing headache disorders, accompanying systemic symptoms, and the overall severity of the acute illness. Notably, headache occurs across the spectrum of COVID-19 severity and is not restricted to patients with severe systemic disease (15–19). However, reported associations have not been entirely consistent across cohorts, likely reflecting differences in study populations, clinical settings, case definitions, and symptom ascertainment. These factors should therefore be regarded as potential epidemiological associations rather than definitive predictors. Detailed study characteristics and findings are summarized in Table 3.

### 2.2 Persistence and longitudinal trajectory and associated factors

Although headache resolves with the acute infection in many patients, a clinically relevant subgroup experiences persistent or recurrent headache for weeks to months after SARS-CoV-2 infection. Longitudinal studies indicate heterogeneous trajectories, ranging from gradual resolution to prolonged persistence or fluctuation over time (19, 20). Persistent headache may represent continuation of headache that began during the acute infection, a new persistent headache phenotype, or exacerbation or transformation of a pre-existing primary headache disorder.

The clinical phenotype of persistent post-COVID headache is likewise heterogeneous. Some patients exhibit migraine-like or tension-type-like features, whereas others may develop a new daily persistent headache (NDPH)-like presentation. Distinguishing *de novo* persistent headache from worsening of a pre-existing migraine or other primary headache disorder can therefore be challenging, particularly in patients with previous headache susceptibility (21, 22).

Factors associated with persistence have varied across studies and may include characteristics of the acute headache, pre-existing headache disorders, demographic factors, and the broader burden of post-COVID symptoms (20, 22–24). Persistent headache is not restricted to patients who experienced severe acute COVID-19, suggesting that chronification cannot be explained solely by the severity of the initial systemic illness.

### 2.3 Potential influence of viral variant and vaccination

The epidemiology and clinical phenotype of COVID-19-associated headache may have varied across successive phases of the pandemic. Observational reports suggest differences in symptom profiles during periods dominated by different SARS-CoV-2 variants; however, direct attribution of these differences to viral genotype remains difficult. Changes in variant circulation have coincided with increasing population immunity, vaccination, previous infections, changes in testing and case ascertainment, and evolving clinical management. Variant-related differences in headache frequency or phenotype should therefore be interpreted as potential associations rather than established causal effects.

Vaccination further complicates the epidemiological context by modifying the risk and severity of SARS-CoV-2 infection while also being independently associated with transient post-vaccination headache (2). Headache occurring after vaccination should therefore be distinguished from headache attributed to SARS-CoV-2 infection. At present, evidence is insufficient to establish consistent variant-specific or vaccination-specific patterns in the occurrence or persistence of COVID-19-associated headache. Prospective longitudinal studies that account for viral variant, vaccination status, previous infection, and baseline headache disorders are needed to clarify these relationships.

### 2.4 Classifications within the ICHD-3 framework

The International Classification of Headache Disorders, 3rd edition (ICHD-3), provides a useful clinical framework for classifying headache occurring during and after SARS-CoV-2 infection (25). Headache developing in close temporal association with acute COVID-19 can generally be considered within the framework of a secondary headache attributed to systemic viral infection when the relevant diagnostic criteria are fulfilled. Establishing the temporal relationship between systemic infection and headache onset is therefore important, particularly because COVID-19-associated headache may exhibit migraine-like or tension-type-like features that overlap with primary headache disorders.

Classification becomes more challenging when headache persists after resolution of the acute systemic infection. In patients whose headache begins during COVID-19 and continues thereafter, the ICHD-3 concept of persistent headache attributed to past systemic viral infection may be applicable (25). However, careful clinical assessment is required to distinguish persistence of an infection-related secondary headache from exacerbation or transformation of a pre-existing migraine, an NDPH-like presentation, or another primary headache disorder. This distinction is particularly relevant in patients with a previous history of migraine or other headache susceptibility.

The temporal thresholds incorporated into existing diagnostic criteria may also be difficult to apply uniformly to the heterogeneous trajectories observed after COVID-19. Accordingly, ICHD-3 classification should be integrated with the individual clinical course rather than interpreted in isolation. Other contributors to chronic or worsening headache, including medication overuse, should also be considered during clinical assessment (26). Further longitudinal phenotyping will be important to determine how persistent post-COVID headache can be most appropriately incorporated into existing headache classification frameworks.

## 3 Clinical phenotypes and characteristic features of COVID-19-associated headache

### 3.1 Core pain features

#### 3.1.1 Pain quality and phenotypic patterning

The quality of headache pain in COVID-19 patients often correlates with their prior headache history. Individuals with a history of migraine predominantly report pulsating or throbbing pain. In contrast, patients without a pre-existing headache disorder more frequently describe a pressing or tightening sensation, often of continuous and unremitting intensity (27). In addition to these history-dependent patterns, COVID-19-associated headache is commonly described as bilateral, with frontal, temporal, or holocranial distribution, and is often moderate to severe in intensity. In some patients, the pain is aggravated by physical activity, coughing, or head movement, and may be accompanied by photophobia, phonophobia, or nausea, further contributing to overlap with established primary headache phenotypes. This distinction is clinically important because it suggests that COVID-19-associated headache is not phenotypically random, but instead shaped by pre-existing headache susceptibility and underlying pain-processing networks. In this sense, SARS-CoV-2 infection may act either as a trigger for “phenotypic copying” of an individual's native headache pattern or as a precipitant of a *de novo* secondary headache phenotype. At the same time, many cases do not fit neatly into conventional migraine or tension-type headache categories, underscoring the hybrid and heterogeneous nature of the clinical phenotype during acute infection.

#### 3.1.2 Intensity, duration, and therapeutic response

Headache intensity in COVID-19 typically ranges from moderate to severe, often described as disabling during the acute phase of infection, as quantified by Visual Analog Scale (VAS) assessments (27–29). Pain may vary from tolerable to incapacitating, with some patients reporting exacerbations reminiscent of severe migraine attacks (28). Duration is highly variable; headaches may last from a few h to several weeks in the acute setting. A significant subset of patients, however, develop persistent headaches lasting months, contributing to the symptom burden of post-acute sequelae of SARS-CoV-2 infection (PASC), or “long COVID” (19, 29, 30). Notably, these headaches can be refractory to conventional analgesics and standard migraine abortive or preventive therapies (31). This variability in duration and treatment response reinforces the view that COVID-19-associated headache encompasses multiple biological subtypes rather than a single uniform clinical entity. From a clinical standpoint, prolonged duration and poor response to standard analgesic strategies may help identify patients at risk of transition from acute infection-related headache to persistent post-viral pain. Importantly, the temporal course of headache during acute SARS-CoV-2 infection is often atypical. Headache may occur early in the disease course and can even be a presenting symptom in some patients. In others, it persists despite improvement in fever or respiratory symptoms, emerges *de novo* in individuals without a prior headache history, or markedly worsens a pre-existing primary headache disorder such as migraine. In a subset of patients, the headache evolves into a prolonged daily pattern resembling persistent post-viral headache or new daily persistent headache. These atypical trajectories further emphasize that COVID-19-associated headache should be viewed as a clinical spectrum rather than a single stereotyped symptom.

### 3.2 Phenotypic heterogeneity

#### 3.2.1 Migraine-like and tension-type-like presentations

The clinical presentation often bifurcates along the lines of pre-existing conditions. Patients with a history of migraine are more likely to experience a migraine-like phenotype, characterized by unilateral, throbbing pain accompanied by photophobia, phonophobia, and/or nausea (21). Conversely, patients without such a history more commonly present with a bilateral, pressing pain consistent with a tension-type headache (TTH) phenotype (27).

Prospective studies have confirmed this dichotomy, identifying distinct clinical clusters. One cluster aligns with migraine features, including aggravation by physical activity, while another is defined by prolonged, pressing pain akin to secondary TTH (32). These observations support the concept that COVID-19-associated headache is phenotypically heterogeneous and may arise through more than one pathophysiological route. The triggering factors and therapeutic responses differ between these clusters, potentially reflecting either the exacerbation of a pre-existing predisposition or *de novo* headache mechanisms related to infection-associated neuroimmune and trigeminovascular perturbations, with direct viral neurotropism remaining less clearly established (33). However, current evidence more consistently supports inflammatory, neuroimmune, and trigeminovascular mechanisms than direct viral neuroinvasion as the principal drivers of most headache presentations. Clinically, distinguishing migraine-like from tension-type-like phenotypes is relevant not only for symptomatic classification, but also for treatment selection and for anticipating which patients may be more likely to follow a persistent or refractory course. More broadly, this overlap with primary headache phenotypes should not obscure the fact that many patients exhibit mixed or atypical features, reinforcing the need for a flexible clinical framework that captures both phenotype and disease context.

#### 3.2.2 Association with chemosensory dysfunction and other neurological features

A distinctive feature of COVID-19-associated headache, particularly the migraine-like phenotype, is its frequent co-occurrence with anosmia (loss of smell) and ageusia (loss of taste) (28, 29). Headache often precedes or coincides with these sensory deficits, suggesting a shared neurobiological mechanism, possibly involving trigeminovascular activation or neuroinflammatory pathways affecting the olfactory system and pain-processing centers (28, 34) (Table 1). From a clinical perspective, this association is especially noteworthy because it may identify a subgroup of patients in whom nasal–olfactory–trigeminal interfaces are more directly involved, potentially contributing to a more distinctive symptom complex and, in some cases, greater persistence of symptoms.

Several mechanisms have been proposed to explain this association. First, SARS-CoV-2-related injury to the olfactory epithelium and associated supporting cells may disrupt chemosensory signaling and indirectly influence trigeminal pathways (35–38). Second, taste disturbance may reflect both peripheral involvement of gustatory structures and secondary impairment of flavor perception resulting from olfactory dysfunction (39–42). Third, local and systemic inflammatory responses—including cytokine-mediated epithelial injury, immune cell infiltration, and glial activation—may provide a shared biological milieu linking chemosensory dysfunction with headache generation and persistence (35, 43–45). Although these mechanisms remain incompletely resolved, the clinical co-occurrence of headache with anosmia and ageusia is one of the most characteristic neurological symptom constellations in COVID-19 and supports the broader view that headache in this setting is embedded within a multisystem neuro-sensory syndrome rather than occurring in isolation. Emerging neurophysiological observations also suggest that SARS-CoV-2 infection may be associated with alterations in brain bioelectrical activity, supporting the possibility of functional central nervous system involvement even when direct structural neuroinvasion remains unproven (46). Although these findings are not specific to headache, they may help explain atypical headache trajectories, altered sensory processing, and symptom persistence in some patients. Clinically, this heterogeneity reinforces the importance of distinguishing *de novo* COVID-19-related headache, exacerbation of pre-existing primary headache disorders, and headache secondary to neurological or vascular complications.

## 4 Mechanistic basis of COVID-19-associated headache

The pathogenesis of headache in COVID-19 is multifactorial, involving interacting inflammatory, neuroimmune, vascular, and systemic processes rather than a single dominant mechanism. Current evidence most consistently supports important roles for inflammatory signaling, endothelial dysfunction, and trigeminovascular activation, whereas direct viral effects on the nervous system remain plausible but less uniformly established. Understanding these mechanisms is important not only for explaining acute symptom generation, but also for clarifying why some patients progress to persistent post-COVID-19-associated headache and why treatment response is often variable.

### 4.1 Inflammatory and neuroimmune mechanisms

Systemic inflammation is a major cornerstone of COVID-19 pathophysiology and likely contributes to its neurological manifestations, including headache. In severe cases, immune dysregulation may lead to a “cytokine storm,” characterized by excessive release of pro-inflammatory cytokines such as interleukin-6 (IL-6), tumor necrosis factor-alpha (TNF-α), and interleukin-1 beta (IL-1β) (47, 48). However, while this severe inflammatory state may explain headache in a subset of critically ill patients, it is unlikely to account for the full clinical spectrum of COVID-19-associated headache. Even outside fulminant cytokine storm states, lower-grade but sustained inflammatory activation may still be sufficient to promote headache through neurovascular and nociceptive pathways.

The inflammatory cascade begins with SARS-CoV-2 infection in the respiratory tract, triggering a systemic immune response. Viral components, including the free S1 subunit of the spike protein, have been proposed to interact with angiotensin-converting enzyme 2 (ACE2) receptors on brain microvascular endothelial cells (BMECs), potentially contributing to blood–brain barrier (BBB) dysfunction and endothelial activation (49–52). Barrier disruption may permit inflammatory mediators, danger signals, and possibly viral products to influence the brain parenchyma and perivascular compartments, thereby promoting microglial activation and neuroinflammation. Experimental studies have shown that spike-related signaling can induce microglial activation and upregulation of inflammatory mediators such as TNF, IFN-β, and IL-6, supporting a biologically plausible link between systemic infection and central pain sensitization (49–52). Although the exact translational relevance of these findings to human headache remains incompletely defined, they provide a credible framework through which peripheral infection may trigger central neuroimmune responses (Figure 1).

**Figure 1:**
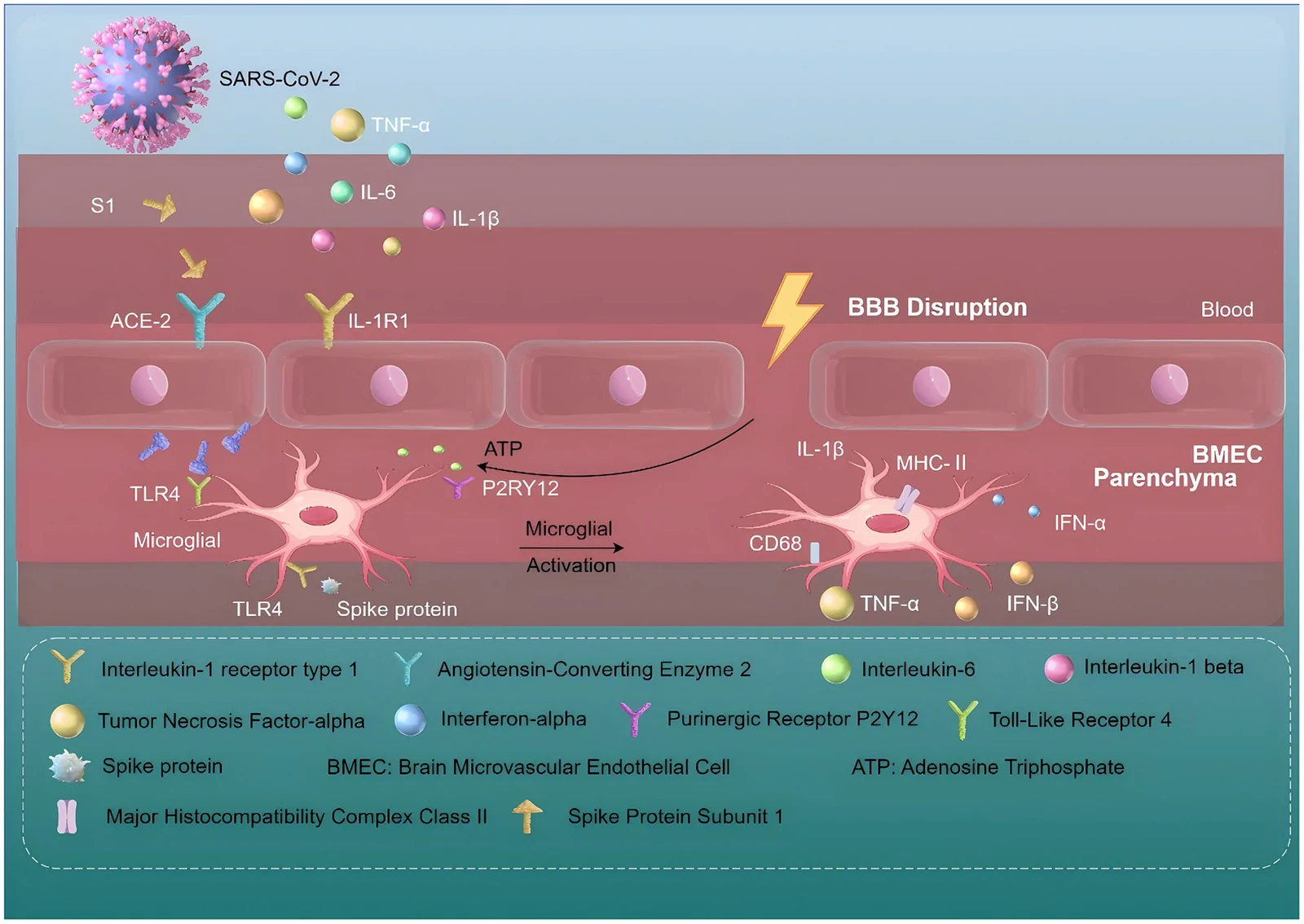
SARS-CoV-2 infection elevates serum pro-inflammatory cytokines such as IL-1β, IL-6, and TNF-α.

The temporal dynamics of key cytokines are also relevant: IL-1β and TNF-α levels surge may rise after infection (Days 1–3), often via NLRP3 inflammasome activation, initiating local inflammatory responses. Because these cytokines are also established mediators of pain biology, their dysregulation in COVID-19 offers a mechanistically coherent explanation for both acute headache and may also contribute tosymptom persistence (Figure 2).

**Figure 2:**
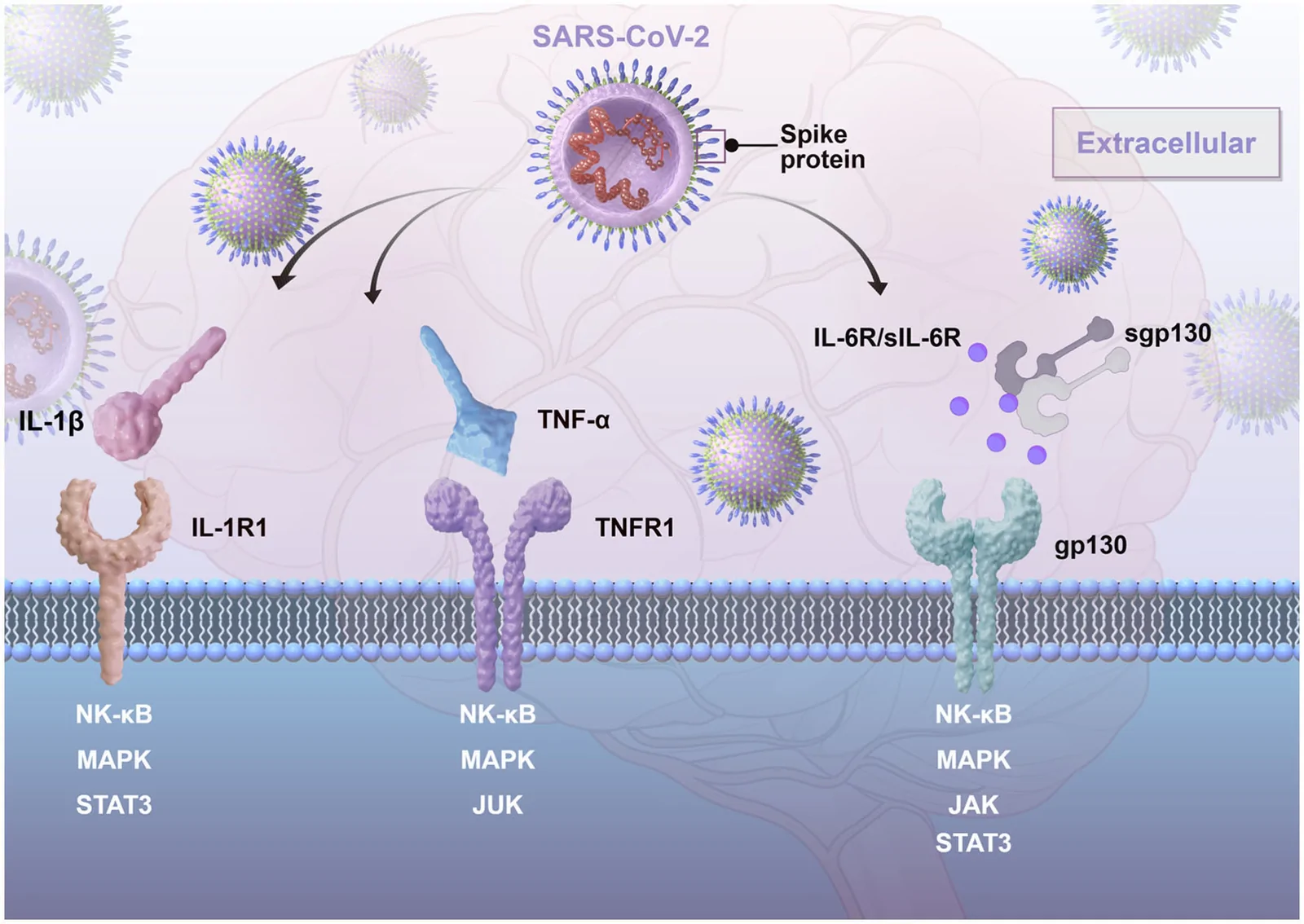
SARS-CoV-2 infection triggers the secretion of IL-1β, TNF-α and IL-6. These pro-inflammatory cytokines bind to their respective receptors to activate NF-κB, STAT3 and MAPK signaling pathways, which synergistically induce neuroinflammation and neuronal hyperexcitability, ultimately driving the pathogenesis of COVID-19-associated headache. SARS-CoV-2, Severe Acute Respiratory Syndrome Coronavirus 2; IL-1β, Interleukin-1β; IL-1R1, Interleukin-1 Receptor Type 1; TNF-α, Tumor Necrosis Factor-alpha; TNFR1, Tumor Necrosis Factor Receptor 1; IL-6, Interleukin-6; IL-6R, Interleukin-6 Receptor; gp130, Glycoprotein 130; NF-κB, Nuclear Factor kappa-light-chain-enhancer of Activated B Cells; STAT3, Signal Transducer and Activator of Transcription 3; MAPK, Mitogen-Activated Protein Kinase.

#### 4.1.1 Persistence may reflect impaired resolution rather than ongoing activation

An additional conceptual framework for persistent COVID-19-associated headache is that symptom chronicity may reflect not only sustained inflammatory or nociceptive activation, but also impaired engagement of endogenous resolution mechanisms. In this view, persistent headache could arise from a failure to restore neuroimmune homeostasis after the acute infectious phase, potentially involving altered autonomic regulation, maladaptive neuroimmune feedback, and insufficient activation of pro-resolving pathways. Related mechanisms may include dysregulation of the inflammatory reflex, persistent sympathetic–parasympathetic imbalance, and prolonged sensitization within trigeminovascular and central pain-processing networks. Although direct evidence for these processes in COVID-19-associated headache remains limited, this perspective complements inflammation-centered models and may help explain why symptoms persist in some individuals despite resolution of the acute infection. It also provides a useful hypothesis-generating framework for future mechanistic studies and for the development of more targeted therapeutic strategies.

#### 4.1.2 IL-1β and inflammasome-linked sensitization

IL-1β may promote both peripheral and central sensitization. It enhances neuronal excitability in the dorsal root ganglion and spinal cord via NF-κB, STAT3 and MAPK/ERK signaling, upregulating pain-related genes (53–56). Critically, it also activates spinal microglia and astrocytes, helping sustain a neuroinflammatory state (57). In headache-relevant tissues, IL-1β may further stimulate cyclooxygenase-2 (COX-2)-dependent prostaglandin E2 (PGE2) production, contributing to meningeal vasodilation and sensitization of trigeminal afferents (58, 59). This positions IL-1β as a plausible mediator linking acute infection-induced inflammation to the initiation of headache pain.

#### 4.1.3 TNF-α and amplification of nociceptive signaling

TNF-α acts primarily through TNF receptor 1 (TNFR1) to promote neuroinflammation and pain-related signaling. TNF-α interacts closely with NF-κB and MAPK pathways, including ERK, JNK and p38. It sensitizes peripheral nociceptors and induces central sensitization in the spinal cord, facilitating long-term potentiation (LTP) of pain signals (59–61). Within the context of COVID-19, TNF-α is therefore best viewed as an amplifier of nociceptive processing rather than an isolated headache-specific mediator.

#### 4.1.4 IL-6 and sustained neuroimmune activation

IL-6 contributes through both classical and trans-signaling pathways. In classical signaling, IL-6 binds membrane-bound IL-6R and gp130, whereas in trans-signaling, IL-6 complexes with soluble IL-6R to activate NF-κB, JAK/STAT3, and MAPK cascades in neurons and glia (62). These pathways may enhance the expression of pain-modulating receptors, including TRPV1, and helps maintain a pro-inflammatory microenvironment (63, 64). Compared with more transient early cytokine responses, sustained IL-6 elevation may be particularly relevant to headache persistence, as it links acute immune activation with prolonged neuroimmune dysregulation.

Taken together, inflammatory and neuroimmune signaling provides the strongest current mechanistic framework for COVID-19-associated headache. Rather than acting independently, cytokines, endothelial activation, glial responses, and nociceptive sensitization likely interact to produce a spectrum ranging from transient acute headache to more persistent and treatment-refractory post-viral pain. Nevertheless, this inflammatory framework should be interpreted alongside epidemiological observations showing that headache is frequently reported in younger patients, women, and individuals with mild-to-moderate COVID-19 (102). This pattern suggests that severe systemic hyperinflammation alone is unlikely to explain all cases and supports the additional consideration of mechanisms such as low-grade neuroimmune activation, peripheral trigeminal sensitization, autonomic dysregulation, and impaired inflammatory resolution.

### 4.2 Olfactory-trigeminal interface, local neuroimmune activation, and putative viral effects

Histopathological and cerebrospinal fluid studies have raised the possibility of SARS-CoV-2 neuroinvolvement; however, direct central nervous system invasion is inconsistently demonstrated and is currently less well supported than inflammatory, neuroimmune, and neurovascular mechanisms as an explanation for headache (12, 15). A more consensus-aligned interpretation is that the olfactory-trigeminal interface may function primarily as a site of local epithelial injury, innate immune activation, cytokine release, and peripheral trigeminal sensitization, particularly in patients with anosmia or other chemosensory disturbances. This model better fits current neuroinfectious and neuroimmune literature, in which local barrier dysfunction and neuroimmune signaling are considered more plausible contributors to headache than widespread productive viral invasion of the brain (24). Regarding viral entry, the primary route is likely retrograde via the olfactory nerve, given the high ACE2 receptor expression in the olfactory epithelium and the strong clinical association with anosmia (48). Alternative routes include hematogenous spread across a disrupted blood-brain barrier (BBB) or via infected immune cells.

A related hypothesis concerns involvement of the trigeminal system. Trigeminal nerve endings in the nasal and oral mucosa, which may be exposed to viral particles and local inflammation, could serve as sites of direct or indirect nociceptive activation (33, 34). Such activation might signal through the trigeminal ganglion and central trigeminal nuclei, thereby contributing to headache generation. This model is particularly attractive in patients whose headache occurs together with anosmia or ageusia, although direct human evidence remains limited.

Beyond direct viral interaction, innate immune signaling within the trigeminal pathway may also be relevant. In a mouse model of trigeminal neuropathic pain, TLR8 was persistently upregulated in trigeminal ganglion neurons, and its activation promoted mechanical allodynia via intracellular Ca^2^^+^ elevation, MAPK phosphorylation, and pro-inflammatory cytokine production (65). Similarly, the chemokine CCL2 and its receptor CCR2 were upregulated in the medullary dorsal horn after trigeminal nerve injury, and blockade of CCR2 alleviated orofacial pain (66). Although these data are not specific to SARS-CoV-2, they support the broader concept that trigeminal neuron–glia and innate immune interactions may amplify or sustain headache in susceptible patients.

### 4.3 Secondary systemic and contextual contributors

Headache in COVID-19 can also be precipitated or exacerbated by systemic complications and broader illness-related stressors. These factors may not be unique to SARS-CoV-2 infection, but they are important because they can intensify headache burden, modify phenotype, and contribute to persistence.

#### 4.3.1 Hypoxia

Severe pulmonary involvement can lead to systemic hypoxemia and cerebral hypoxia. This may disrupt cerebral metabolism and autoregulation, increase oxidative stress, and trigger headache directly. In this context, headache may reflect not only inflammatory activation but also impaired oxygen delivery and altered neurovascular homeostasis.

#### 4.3.2 Endothelial dysfunction and coagulopathy

SARS-CoV-2 infection causes endotheliitis and a pro-thrombotic state. Microvascular injury and microthrombi can impair cerebral perfusion, leading to ischemic pain or contributing to conditions like cerebral venous sinus thrombosis, which presents with headache (15, 33). Among secondary systemic mechanisms, endothelial dysfunction is particularly important because it offers a plausible bridge between inflammation, vascular dysregulation, and trigeminovascular activation.

#### 4.3.3 Psychosocial stress, sleep disruption, and affective burden

The pandemic and the illness experience itself have been major sources of stress, anxiety, sleep disturbance, and depression, all of which are well-established triggers or amplifiers of primary headache disorders (12). These factors can worsen pre-existing headache, facilitate *de novo* headache, and lower the threshold for pain chronification through hypothalamic–pituitary–adrenal axis dysregulation and impaired endogenous pain control. In persistent post-COVID-19-associated headache, such psychosocial and behavioral factors likely interact with biological mechanisms rather than acting as purely external modifiers.

Pain and depression frequently co-occur in chronic pain conditions, including long COVID. In a neuropathic pain model, CCL2 and CCR2 were upregulated in the nucleus accumbens, a key brain region for reward and mood regulation, and their activation promoted both pain hypersensitivity and depressive behaviors via ERK-dependent NR2B phosphorylation (67). While extrapolation to COVID-19 should remain cautious, these findings highlight the plausibility of shared molecular pathways linking persistent pain and affective symptoms in PASC.

### 4.4 Integrated model: from acute headache to persistent post-COVID pain

The trigeminovascular system (TVS) likely functions as the principal final common pathway through which diverse pathophysiological mechanisms converge to generate headache in COVID-19. As the major nociceptive network underlying many primary and secondary headache disorders, the TVS can be activated through several interrelated routes during SARS-CoV-2 infection. Inflammatory mediators such as IL-1β, TNF-α, IL-6, and prostaglandins may directly sensitize trigeminal nerve endings and meningeal vessels. Endothelial dysfunction, ischemia, and hypoxia may further enhance neurovascular irritability. At least in some patients, local mucosal inflammation or putative trigeminal pathway involvement may provide additional peripheral input. Persistent neuroinflammation, psychological stress, and repeated nociceptive signaling may then lower the threshold for central sensitization and amplify pain transmission.

Activation of the TVS promotes release of vasoactive neuropeptides such as calcitonin gene-related peptide (CGRP) and substance P, perpetuating inflammation, vasodilation, and nociceptive signaling to the trigeminal nucleus caudalis and higher cortical centers (33, 34) (Figure 3). Within this framework, acute COVID-19 headache may be viewed as the clinical expression of convergent inflammatory, neuroimmune, neurovascular, and peripheral trigeminal inputs, whereas persistent post-COVID-19-associated headache may reflect incomplete resolution of these processes in susceptible individuals. This integrative model helps explain why COVID-19-associated headache may resemble migraine in some patients, tension-type headache in others, and chronic daily or persistent post-viral headache in a subset with prolonged symptoms. Within this framework, acute COVID-19 headache may be viewed as the clinical expression of convergent inflammatory, neuroimmune, neurovascular, and peripheral trigeminal inputs, whereas persistent post-COVID-19-associated headache may reflect incomplete resolution of these processes in susceptible individuals. It also provides a biologically plausible rationale for therapies targeting trigeminovascular signaling, including CGRP-directed approaches, although current evidence remains insufficient to define which patient subgroups are most likely to benefit.

**Figure 3:**
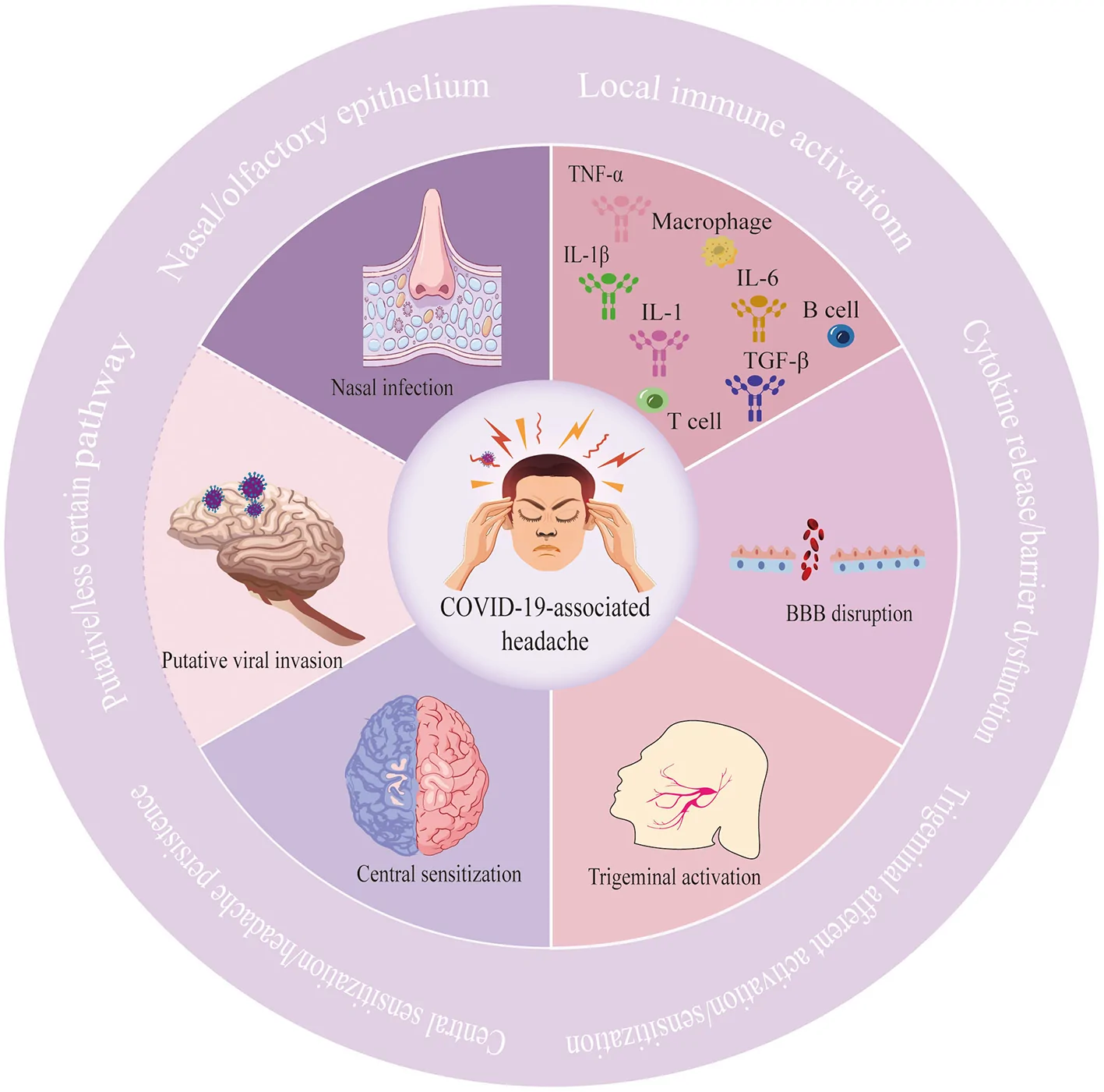
Proposed mechanistic model of COVID-19-associated headache. Current evidence most strongly supports inflammatory, neuroimmune, endothelial, and trigeminovascular mechanisms. Direct viral neuroinvasion remains hypothetical and less consistently supported.

Overall, the mechanistic landscape of COVID-19-associated headache is best understood as a layered model in which systemic inflammation, neuroimmune activation, vascular dysfunction, trigeminal sensitization, and host susceptibility interact dynamically. Sex-specific neuroendocrine-immune influences may further modulate this process, potentially contributing to interindividual differences in headache susceptibility, severity, and persistence. This framework is particularly useful because it accommodates both acute symptom generation and the transition to persistent post-COVID-19-associated headache. Taken together, COVID-19-associated headache is best conceptualized as a dynamic continuum rather than a single-pathway phenomenon, in which the interaction between biological triggers and host vulnerability determines whether headache remains transient or evolves into persistent post-COVID pain.

### 4.5 Sex-related susceptibility to COVID-19-associated headache

An additional issue that warrants specific consideration is the apparent female predominance of headache in both acute COVID-19 and post-COVID conditions, as highlighted in Figure 4. Although headache disorders are generally more common in women, this pattern in COVID-19 may also reflect sex-specific biological mechanisms rather than baseline epidemiologic differences alone. Women typically mount stronger innate and adaptive immune responses than men, which may enhance viral clearance but may also increase cytokine production, neuroimmune activation, and inflammatory pain signaling during and after SARS-CoV-2 infection.

**Figure 4:**
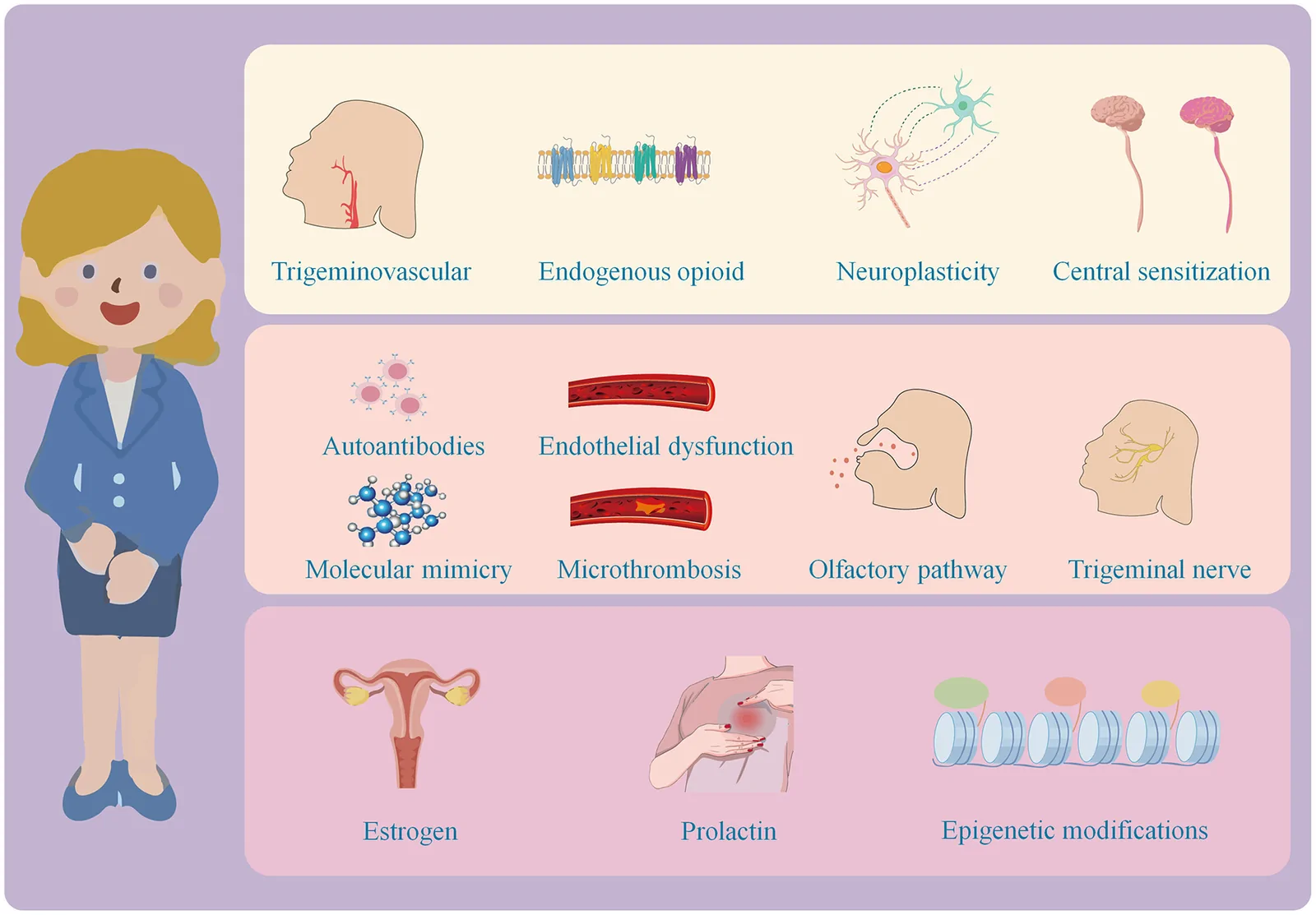
The potential mechanisms underlying the higher prevalence of headache in females compared to males were neuroinflammation and pain pathways, immune & vascular mechanisms and hormonal & epigenetic regulation.

Sex hormones may further modulate headache susceptibility through effects on the trigeminovascular system. Estrogen has been implicated in the regulation of calcitonin gene-related peptide (CGRP) signaling, trigeminal excitability, and nociceptive sensitization, while prolactin and mast cell activation may further amplify neurogenic inflammation and pain transmission. These interactions provide a biologically plausible basis for a lower threshold for headache generation and persistence in women, particularly in individuals with pre-existing migraine susceptibility.

Accordingly, the sex difference suggested in Figure 4 is best understood as the result of converging neuroendocrine, immune, and trigeminovascular influences that may increase both the likelihood and persistence of COVID-19-associated headache in female patients. This interpretation helps connect the conceptual framework shown in the figure with the clinical observation that women appear to bear a disproportionate burden of headache in long COVID.

## 5 COVID-19 vaccine-associated headache

Headache is a common adverse event following COVID-19 vaccination, typically occurring within hours to days after injection, presenting as moderate to severe bilateral pressing or pulsating pain, sometimes accompanied by migrainous features such as photophobia, phonophobia, and nausea, and usually resolving within 48–72 hours with or without simple analgesics (2, 68). In a minority of cases, however, headache may persist or recur over subsequent days to weeks. This prolonged pattern appears to be more common and clinically more complex in individuals with pre-existing primary headache disorders, particularly migraine, who are at higher risk for both acute and prolonged post-vaccination headache and more often experience exacerbations of their usual headache phenotype with greater intensity, longer duration, and more pronounced migrainous features (68). By contrast, individuals without a prior headache history more commonly report milder, shorter-lasting headaches, although some may develop new-onset persistent pressing or stabbing pain requiring specialist evaluation (2, 32). Overall, these findings suggest that post-vaccination headache is generally benign and self-limited, but its phenotype is influenced by baseline headache susceptibility; severe, delayed, progressive, or atypical headache, especially when accompanied by focal neurological deficits, visual symptoms, seizures, or marked systemic illness, should prompt assessment for secondary causes, including rare thrombotic complications in appropriate clinical contexts.

## 6 Clinical management of COVID-19-associated headache

Management of COVID-19-associated headache should be guided by headache phenotype, temporal course, associated neurological or systemic features, and the need to distinguish exacerbation of a primary headache disorder from secondary headache related to infection or its complications. In the acute phase, treatment is usually supportive and symptom-directed, with acetaminophen or NSAIDs as first-line options, selected according to clinical severity, comorbidities, and medication safety profile. Refractory or atypical headaches require both escalation of symptom control and reassessment for secondary causes, including meningoencephalitis, cerebral venous thrombosis, intracranial hypertension, and other neurological or vascular complications. In selected patients, migraine-specific therapies, short courses of corticosteroids, or individualized preventive agents may be considered, although evidence remains limited. For persistent post-COVID-19-associated headache, management generally shifts to a phenotype-informed chronic care model combining preventive medications with non-pharmacological measures such as sleep regulation, hydration, graded exercise, cognitive-behavioral therapy, relaxation strategies, and physical rehabilitation. Because persistent headache often coexists with fatigue, cognitive symptoms, sleep disturbance, anxiety, and depression, multidisciplinary care and psychological support are frequently essential. Telemedicine can facilitate follow-up, treatment adjustment, education, and remote behavioral interventions in appropriately selected patients, although it should complement rather than replace in-person evaluation when red flags or secondary headache are suspected. Treatment of COVID-19-associated headache remains largely phenotype-driven, as direct evidence for disease-specific interventions is limited. A practical summary of acute, preventive, and adjunctive management options—including medication-overuse management, CGRP-targeted therapies, onabotulinumtoxinA, and greater occipital nerve blockade in selected refractory cases—is provided in Table 4.

**Table 4:** Current therapeutic options for COVID-19-associated headache and level of supporting evidence.

Intervention	Potential clinical role	Evidence specific to COVID-19-associated headache	Practical interpretation
Simple analgesics/acetaminophen	Acute symptomatic relief	Low–moderate (2, 12, 15, 72)	Common first-line option in acute infection; evidence mostly based on routine clinical use rather than COVID-specific trials
NSAIDs	Acute symptomatic relief	Low–moderate (2, 12, 15, 72)	Frequently used in acute COVID-related headache if no contraindications
Triptans	Migraine-like acute attacks or exacerbations	Low (2, 15, 19, 30)	May be considered in selected patients with migraine-like phenotype; direct COVID-specific evidence remains limited
Conventional preventive therapies for migraine-like headache	Persistent/post-COVID-19-associated headache with migraine phenotype	Low (2, 15, 19, 30)	Often extrapolated from standard migraine management
CGRP-targeted therapies	Refractory persistent migraine-like post-COVID-19-associated headache	Low (2, 15, 19, 20, 30, 99)	Supported mainly by case reports/small series and indirect extrapolation from migraine prevention recommendations for CGRP-targeted therapies
OnabotulinumtoxinA	Chronic frequent or chronic migraine-like post-COVID-19-associated headache	Low (2, 15, 19, 20, 30, 100)	May be considered in selected refractory cases; direct evidence remains limited, but use is supported indirectly by its established role in chronic migraine
Greater occipital nerve blockade	Refractory cases with occipital/cervicogenic or migraine-like features	Very low (2, 15, 20, 30, 101)	Adjunctive or bridge intervention in selected patients; rationale is mainly extrapolated from migraine/refractory headache literature
Medication-overuse management	Frequent/daily headache with repeated acute medication use	Indirect but clinically important (2, 15, 19, 20, 25, 26, 30)	Standard MOH principles should be applied when overuse is suspected; MOH should be assessed according to ICHD-3 criteria, with reduction or withdrawal of overused acute medications when appropriate
Sleep optimization/hydration/routine regulation	Supportive symptom reduction	Low (2, 15, 20, 30)	Reasonable low-risk supportive measures
Psychological interventions (e.g., CBT, stress management, relaxation)	Headache persistence with stress, anxiety, central sensitization, maladaptive coping	Low (8, 10, 20, 23, 30)	Mainly extrapolated from chronic headache and chronic pain literature
Physical therapy	Cervical, myofascial, or postural contributors	Low (20, 23, 30)	May help in selected patients with musculoskeletal involvement
Multidisciplinary rehabilitation	Complex long COVID with headache plus fatigue/cognitive/autonomic symptoms	Low direct/moderate indirect (8, 10, 20, 23, 30)	Particularly relevant when headache occurs as part of broader post-COVID disability

## 7 Future directions and clinical priorities

Despite substantial progress, important gaps remain in the understanding of COVID-19-associated headache. Future research should clarify the sources of variability in prevalence and phenotype across populations and study designs, and move from descriptive associations toward mechanism-informed stratification. Current evidence supports a multifactorial pathophysiology involving inflammatory, neuroimmune, endothelial, and trigeminovascular pathways, but their relative contributions, interactions, and phenotype-specific relevance remain uncertain. Longitudinal, deeply phenotyped cohorts are needed to define headache trajectories, identify predictors of persistence and chronification, and clarify the transition from acute infection-related headache to chronic pain states. Clinical priorities include improving diagnostic distinction between COVID-19-triggered headache, exacerbation of pre-existing primary headache disorders, and secondary headache due to neurological or vascular complications; refining classification frameworks; and evaluating biomarkers, neuroimaging, and digital phenotyping tools for better stratification. Therapeutically, stronger evidence is needed for both acute and persistent headache, including trials of migraine-specific, anti-inflammatory, and multidisciplinary interventions. In parallel, telemedicine and digital health tools may help expand access, support longitudinal monitoring, and improve continuity of care. Overall, future progress will depend on integrating epidemiology, mechanisms, diagnosis, and treatment within a more coherent, phenotype-sensitive framework.

## 8 Conclusion

COVID-19-associated headache is now recognized as a common and clinically significant neurological manifestation of SARS-CoV-2 infection, spanning a spectrum from self-limited acute headache to persistent post-COVID forms that contribute meaningfully to long COVID burden. It shows substantial phenotypic and epidemiological heterogeneity, with higher susceptibility reported in younger adults, women, and individuals with pre-existing primary headache disorders. Current evidence supports a multifactorial pathophysiology involving systemic inflammation, neuroimmune and trigeminovascular activation, and endothelial or vascular dysfunction, while direct viral neuroinvasion remains less consistently supported. Most acute cases can be managed supportively, whereas persistent headache often requires individualized, multidisciplinary care. Moving forward, the field should prioritize longitudinal and translational studies to define natural history, identify drivers of persistence, refine diagnostic classification, and test targeted therapies in stratified populations. Beyond its immediate clinical relevance, COVID-19-associated headache also offers a useful model for understanding post-infectious pain syndromes and improving preparedness for future infectious outbreaks.
